# Supplementary material for: Antiarrhythmic Properties of Elsholtzia ciliata Essential Oil on Electrical Activity of the Isolated Rabbit Heart and Preferential Inhibition of Sodium Conductance
Source: Biomolecules. 2020 Jun 23;10(6):948. doi: 10.3390/biom10060948 (PMC7356736; doi:10.3390/biom10060948)
Supplement: Supplementary file 1 [file biomolecules-10-00948-s001.pdf]

Supplementary Materials

Table S1. Chemical composition of essential oil obtained by hydrodistillation from *E. ciliata* dried herb using GC-MS analysis

Compounds	Retention index	Composition (%)
Eucalyptol	963	0.05
Cyclohexene, 2-ethenyl-1,3,3-trimethyl	1011	0.15
2-propenoic acid, 2-methyl-, ethenyl ester	1053	0.06
Elsholtzia ketone	1066	14.58
Furane-2-carboxaldehyde, 5-(nitrophenoxymethyl)-	1079	0.43
(-)-1R-8-Hydroxy-p-menth-4-en-3-one	1110	0.08
Dehydroelsholtzia ketone	1117	78.28
Beta-Bourbonene	1152	0.57
Isocaryophyllene	1166	0.57
Beta-Cubebene	1170	0.06
Ledene	1174	0.05
Alpha-Caryophyllene	1180	1.84
Alpha-Cubebene	1186	0.02
Naphthalene	1190	0.13
Germacrene D	1192	0.24
Trans-alpha-Bergamotene	1197	0.55
Alpha-Farnesene	1202	0.66
Gamma-Cadinene	1205	0.15
Delta-Cadinene	1208	0.28
Caryophyllene oxide	1224	0.21
Nonane	1243	0.05
3-Tetradecen-5-yne, (Z)-	1268	0.05
Palmitic acid	1275	0.16
Phytol	1286	0.06
Methyl (Z)-5,11,14,17-eicosatetraenoate	1289	0.61
2,6-octadiene, 2,7-dimethyl-	1294	0.08
Sesquiterpenes		4.99
Oxygenated monoterpenes		0.05
Oxygenated sesquiterpenes		0.21
Ketones		92.86
Others		1.86
Total		99.97

Data according to Pudziuvelyte et. al. [5]

Table S2. The effect of *E. ciliata* essential oil on the AP changes in the rabbit heart.

Conc. (μL/mL)	AT (ms) (ms)	dV/dt_{max} (V/s)	APA (mV)	APD20 (ms)	APD50 (ms)	APD90 (ms)	RP (mV)
Atrial-pacing							
Control	104.7 ± 3.5	102.5 ± 9.2	110.2 ± 1.5	91.9 ± 2.4	132.8 ± 3.4	157.2 ± 2.8	-80.7 ± 0.2
0.01	106.5 ± 3.7*	92.8 ± 9.0*	108.2 ± 1.6	86.7 ± 3.6*	129.8 ± 3.7*	155.6 ± 2.9	-80.3 ± 0.3
0.03	110.2 ± 4.7*	77.4 ± 8.4*	106.3 ± 1.8*	80.1 ± 4.4*	125.7 ± 4.7*	152.9± 3.8*	-80.1 ± 0.3*
0.1	121.1 ± 6.0*	54.1 ± 9.6*	101.3 ± 1.9*	66.9 ± 4.2*	110.2 ± 5.2*	140.7 ± 4.3*	-79.8 ± 0.3*
0.3	196.3 ± 10.4*	28.9 ± 4.3*	81.0 ± 3.7*	48.8 ± 3.7*	83.2 ± 4.2*	114.6 ± 3.8*	-78.3 ± 0.3*
Washout	119.9 ± 6.1#	58.5 ± 7.7#	105.0 ± 0.7#	74.2 ± 7.2#	127.6 ± 10.6#	158.5 ± 10.4#	-80.2 ± 0.3#
Endo-pacing							
Control	32.3 ± 3.1	95.8 ± 11.2	109.8 ± 1.2	92.1 ± 2.3	133.1 ± 3.4	157.7 ± 2.5	-80.8 ± 0.2
0.01	33.2 ± 3.2*	88.4 ± 10.9*	109.7 ± 1.8	86.9 ± 2.5*	130.3 ± 3.4*	156.0 ± 2.7	-80.4 ± 0.2
0.03	35.4 ± 3.5*	76.7 ± 11.6*	107.2 ± 1.7*	80.5 ± 4.0*	125.9 ± 4.5*	153.1 ± 3.6*	-79.9 ± 0.1*
0.1	43.9 ± 6.2*	58.9 ± 11.5*	103.4 ± 1.9*	66.8 ± 4.3*	110.9 ± 5.7*	140.7 ± 4.9*	-79.6 ± 0.1*
0.3	70.1 ± 6.4*	32.3 ± 6.8*	88.7 ± 2.7*	57.8 ± 3.2*	95.0 ± 4.6*	127.5 ± 4.4*	-78.8 ± 0.2*
Washout	43.9 ± 3.2#	62.9 ± 8.4#	105.6 ± 0.7#	75.5 ± 4.7#	130.0 ± 6.1#	162.9 ± 4.4#	-80.6 ± 1.5#
Epi-pacing							
Control	31.6 ± 2.8	86.7 ± 5.5	109.8 ± 1.6	91.0 ± 2.3	130.8 ± 2.3	156.3 ± 1.8	-80.4 ± 0.4
0.01	32.1 ± 2.9*	79.8 ± 5.4*	106.1 ± 2.3	87.8 ± 3.3	129.3 ± 2.7	156.1 ± 2.1	-79.7 ± 0.5
0.03	33.9 ± 3.2*	70.8 ± 3.9*	103.8 ± 1.6*	82.1 ± 4.3*	125.1 ± 4.1	152.8 ± 3.0	-79.6 ± 0.4
0.1	40.8 ± 4.3*	53.0 ± 5.2*	98.9 ± 2.9*	69.3 ± 4.2*	112.1 ± 4.7*	142.5 ± 4.1*	-79.4 ± 0.4*
0.3	63.4 ± 5.8*	29.7 ± 4.2*	90.9 ± 3.2*	52.0 ± 3.0*	88.8 ± 3.3*	122.9 ± 2.3*	-78.6 ± 0.8*
Washout	40.4 ± 4.3#	60.8 ± 7.5#	104.2 ± 0.9#	79.1 ± 4.3#	130.4 ± 4.6#	163.3 ± 3.0#	-80.2 ± 0.3#

Tyrode solution without *E. ciliata*, Control and Washout; Conc.: concentration; AT: activation time; dV/dt_{max}: the maximum value of the first time derivative of the AP upstroke; APA: action potential (AP) amplitude; APD20, APD50 and APD90: AP duration at 20%, 50% and 90% levels of repolarization, respectively; RP: resting membrane potential; Note: values are the mean ± S.E.M., n = 7 for each; * $p < 0.05$ for *E. ciliata* vs. control; # $p < 0.05$ for washout vs. *E. ciliata* 0.3 μL/mL concentration.

Table S3. The efficacy of the *E. ciliata* action on OAP changes in the rabbit heart.

Conc. (μL/mL)	ΔF/F (%)	AT50 (ms)	UD20-80 (ms)	OAPD20 (ms)	OAPD50 (ms)	OAPD90 (ms)
Atrial-pacing						
Control	11.4 ± 2.7	115.9 ± 2.4	7.2 ± 0.6	97.5 ± 1.84	136.3 ± 2.3	160.5 ± 2.5
0.01	11.9 ± 2.5	120.7 ± 4.1	7.4 ± 0.8	92.0 ± 2.3*	131.7 ± 3.0*	156.7 ± 2.5*
0.03	11.7 ± 2.3	125.0 ± 5.0*	7.7 ± 0.8	84.9 ± 3.0*	126.4 ± 3.6*	153.4 ± 3.1*
0.1	10.8 ± 1.9	138.7 ± 6.4*	8.7 ± 0.7*	70.6 ± 3.9*	111.8 ± 4.4*	142.5 ± 4.0*
0.3	8.6 ± 1.7	204.9 ± 9.2*	17.7 ± 2.6*	62.8 ± 2.6*	94.0 ± 3.7*	129.0 ± 2.6*
Washout	10.1 ± 1.8#	135.4 ± 5.3#	9.2 ± 0.9#	80.4 ± 1.5#	130.3 ± 3.9#	161.3 ± 4.1#
Endo-pacing						
Control	11.3 ± 2.7	30.2 ± 2.8	10.6 ± 1.6	98.4 ± 1.9	135.9 ± 2.2	160.6 ± 2.5
0.01	11.9 ± 2.5	31.5 ± 2.8*	10.9 ± 1.7	92.4 ± 2.6*	131.4 ± 3.0*	157.1 ± 2.8*
0.03	11.6 ± 2.2	33.6 ± 2.9*	11.3 ± 1.8	86.4 ± 3.0*	126.7 ± 3.5*	153.9 ± 3.1*
0.1	10.6 ± 1.9	40.2 ± 4.3*	13.0 ± 1.9*	73.0 ± 3.7*	112.5 ± 4.2*	144.4 ± 3.7*
0.3	8.4 ± 1.7	67.4 ± 10.2*	19.4 ± 2.0*	65.5 ± 2.8*	96.1 ± 3.4*	134.3 ± 2.9*
Washout	10.0 ± 1.8#	39.2 ± 2.7#	13.5 ± 2.2#	82.7 ± 1.6#	130.7 ± 3.8#	164.7 ± 4.1#
Epi-pacing						
Control	11.3 ± 2.7	39.8 ± 2.5	8.9 ± 0.9	97.2 ± 1.3	135.2 ± 2.0	160.5 ± 2.5
0.01	11.8 ± 2.5	41.2 ± 2.4*	9.3 ± 1.0	91.8 ± 2.4*	131.0 ± 2.8*	156.7 ± 2.5*
0.03	11.6 ± 2.2	43.1 ± 2.5*	9.7 ± 1.2	85.6 ± 2.7*	126.1 ± 3.4*	153.4 ± 3.1*
0.1	10.6 ± 1.9	49.1 ± 2.5*	11.6 ± 1.5*	72.9 ± 3.4*	112.7 ± 4.1*	142.5 ± 3.9*
0.3	8.4 ± 1.7	64.8 ± 5.7*	17.0 ± 2.2*	65.1 ± 2.8*	95.8 ± 3.6*	128.9 ± 2.6*
Washout	9.9 ± 1.8#	48.3 ± 2.8#	12.3 ± 1.9#	82.5 ± 1.9#	130.3 ± 3.9#	161.3 ± 4.1#

Tyrode solution without *E. ciliata*, Control and Washout; Conc.: concentration; ΔF/F: voltage-sensitive fraction of fluorescence; AT50: activation time at 50% level of depolarization of the OAP; UD20-80: OAP upstroke duration between 20% and 80% of depolarization; OAPD20, OAPD50 and OAPD90: OAP duration at 20%, 50% and 90% levels of repolarization, respectively; Note: values are the mean ± S.E.M., n = 6 for each; * $p < 0.05$ for *E. ciliata* vs. control; # $p < 0.05$ for washout vs. *E. ciliata* of 0.3 μL/mL concentration.

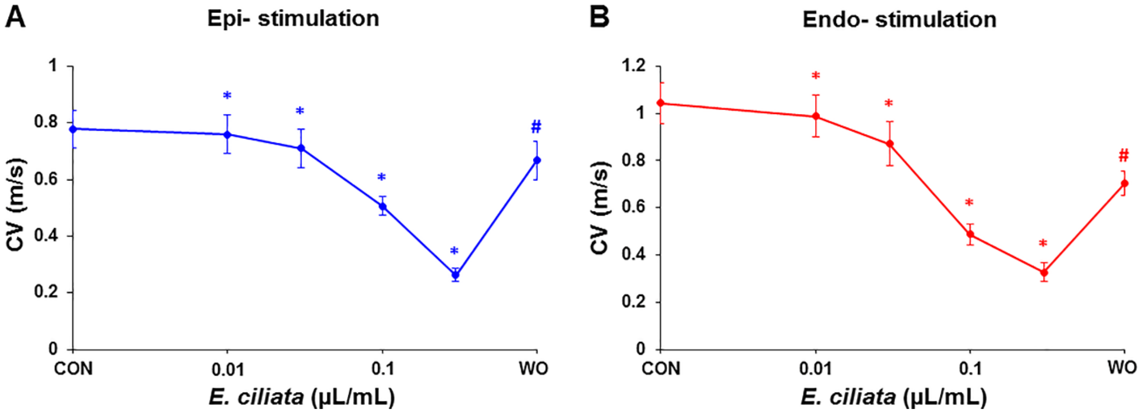

Figure S1. Dependence of the conduction velocity on the *E. ciliata* concentrations. (A-B) Steady-state level of conduction velocity measurements at epi- and endo-cardial stimulation (300-ms period), respectively. Conduction velocity was taken under control conditions (CON) and at the end of 10-min with 0.01, 0.03, 0.1 and 0.3 μL/mL concentrations of the *E. ciliata*, followed by washout period (WO) (* $p < 0.05$ for *E. ciliata* vs. control; $^{\#}p < 0.05$ for washout vs. *E. ciliata* at 0.3 μL/mL concentration; n = 5-6 for each).

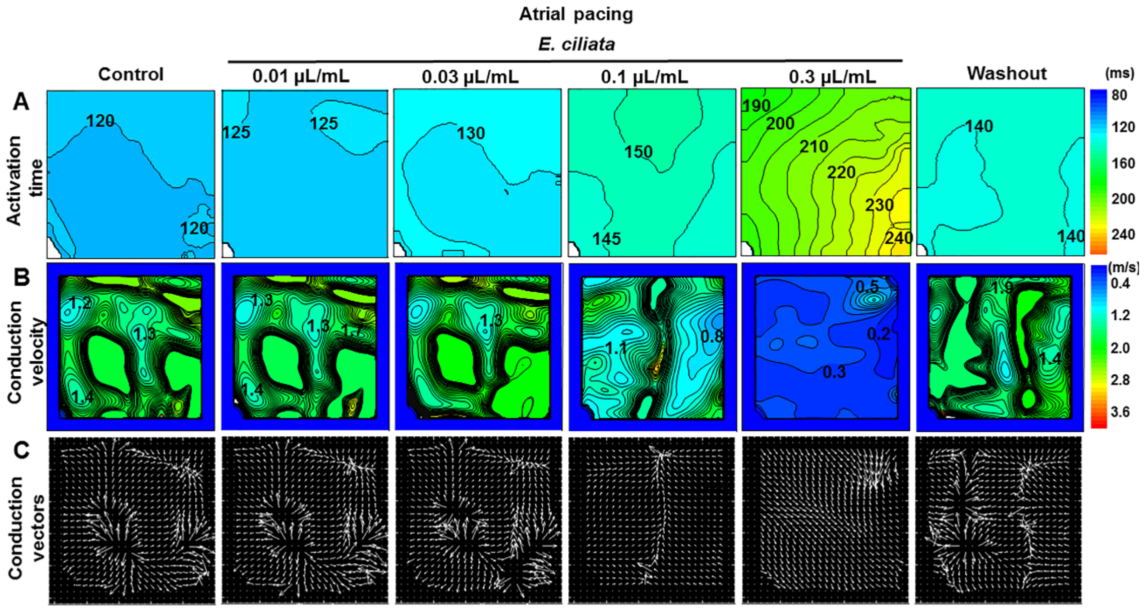

Figure S2. Atrial stimulation: Optical maps revealing *E. ciliata* effects on electrical activation process in the heart. Pacing period 300-ms. (**A**) Activation time (in ms), (**B**) Conduction velocity (in m/s), and (**C**) Conduction vectors maps in control and at 0.01, 0.03, 0.1, and 0.3 μL/mL concentrations of the *E. ciliata*, followed by washout period. The interval between isochrones is 5 ms for the activation time, 0.1 m/s for the conduction velocity. The direction of movement of the activation wave is from blue-green to red. Of note, during atrial stimulation, almost simultaneous activation (~ in 2 ms) of the endocardium via Purkinje fibres occurs and, under such circumstances, transmural propagation is dominating comparing with the lateral excitation wave propagation as presented in the conduction velocity maps. Mean values of conduction velocity under atrial stimulation are uninformative and were not calculated.

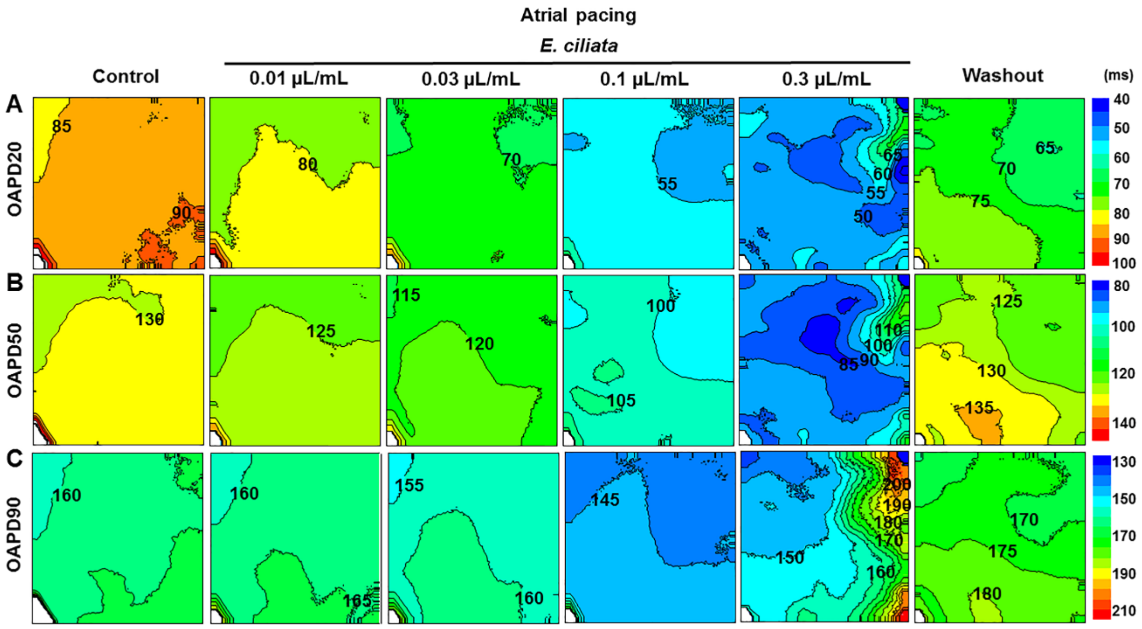

Figure S3. Atrial stimulation: Changes in OAP duration maps induced by *E. ciliata*. Pacing period 300-ms. (**A**) OAPD20, (**B**) OAPD50, and (**C**) OAPD90 maps in control and at 0.01, 0.03, 0.1, and 0.3 µL/mL concentrations of the *E. ciliata*, followed by washout period. OAPD20, OAPD50, and OAPD90 maps calculated at 20%, 50%, and 90% of repolarization, respectively. The interval between isochrones is 5 ms. Other notations are the same as in Figure S2.

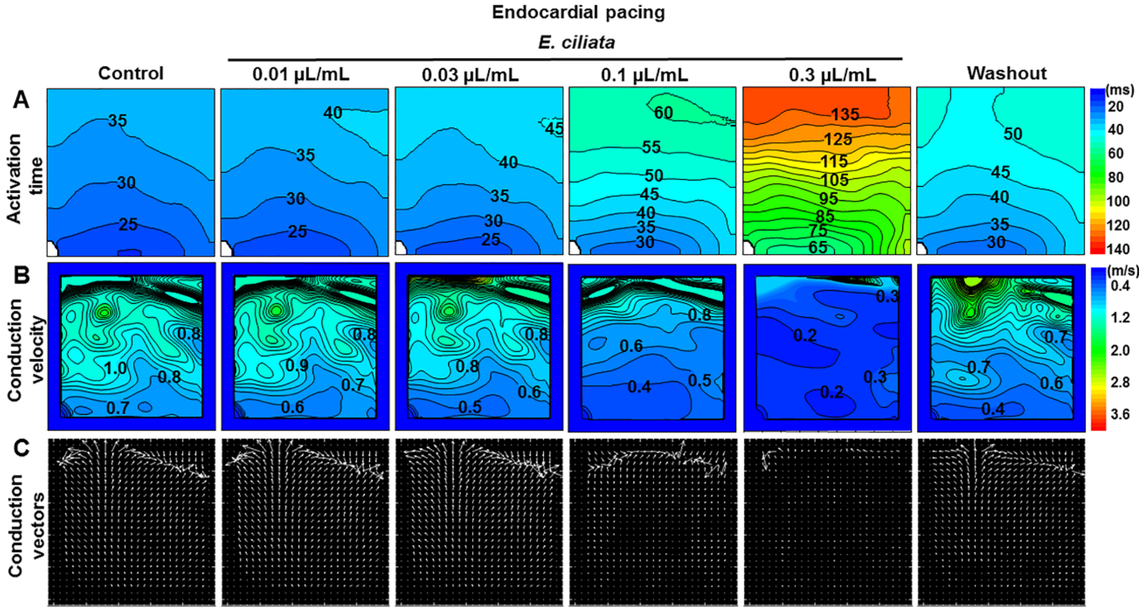

Figure S4. Endocardial stimulation: Optical maps revealing *E. ciliata* effects on electrical activation process in the heart. (**A**) Activation time, (**B**) Conduction velocity, and (**C**) Conduction vector maps in control and at 0.01, 0.03, 0.1, and 0.3 µL/mL concentrations of the *E. ciliata*, followed by washout period. Stimulation electrode was located close to the apex. Other notations are the same as in Figure S2.

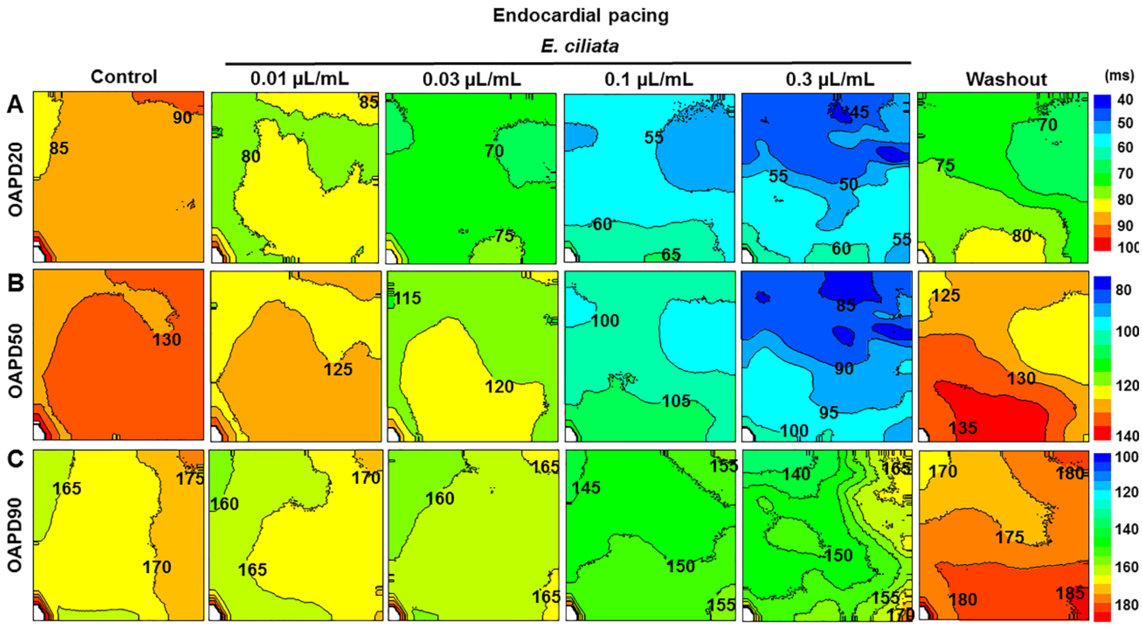

Figure S5. Endocardial stimulation: Changes in OAP duration maps induced by *E. ciliata*. (A) OAPD20, (B) OAPD50, and (C) OAPD90 maps in control and at 0.01, 0.03, 0.1, and 0.3 µL/mL concentrations of the *E. ciliata*, followed by washout period. Other notations are the same as in Figure S3.
